# Metabolomic Fingerprinting of Potato Cultivars Differing in Susceptibility to *Spongospora subterranea* f. sp. *subterranea* Root Infection

**DOI:** 10.3390/ijms21113788

**Published:** 2020-05-27

**Authors:** Moleboheng Lekota, Kehumile J. Modisane, Zeno Apostolides, Jacquie E. van der Waals

**Affiliations:** 1 Department of Plant and Soil Sciences, Forestry and Agricultural Biotechnology Institute, University of Pretoria, Private Bag X20, Hatfield, Pretoria 0028, South Africa; lebolek@yahoo.com; 2 Department of Crop Science, National University of Lesotho, Roma 180, Lesotho; 3 Department of Biochemistry, Genetics and Microbiology, University of Pretoria, Private Bag X20, Hatfield, Pretoria 0028, South Africa; kehumile.modisane@gmail.com (K.J.M.); zeno.apostolides@up.ac.za (Z.A.)

**Keywords:** potato roots, chromatogram, antimicrobial, phytoalexins, UPLC-MS, phytochemicals, root exudates, powdery scab

## Abstract

Plants defend themselves from pathogens by producing bioactive defense chemicals. The biochemical mechanisms relating to quantitative resistance of potato to root infection by *Spongospora subterranea* f. sp. *subterranea* (Sss) are, however, not understood, and are not efficiently utilized in potato breeding programs. Untargeted metabolomics using ultra-performance liquid chromatography coupled with quadrupole time-of-flight mass spectrometry (UPLC-Q-TOF/MS) was used to elucidate the biochemical mechanisms of susceptibility to Sss root infection. Potato roots and root exudate metabolic profiles of five tolerant cultivars were compared with those of five susceptible cultivars, following Sss inoculation, to identify tolerance-related metabolites. Comparison of the relative metabolite abundance of tolerant versus susceptible cultivars revealed contrasting responses to Sss infection. Metabolites belonging to amino acids, organic acids, fatty acids, phenolics, and sugars, as well as well-known cell wall thickening compounds were putatively identified and were especially abundant in the tolerant cultivars relative to the susceptible cultivars. Metabolites known to activate plant secondary defense metabolism were significantly increased in the tolerant cultivars compared to susceptible cultivars following Sss inoculation. Root-exuded compounds belonging to the chemical class of phenolics were also found in abundance in the tolerant cultivars compared to susceptible cultivars. This study illustrated that Sss infection of potato roots leads to differential expression of metabolites in tolerant and susceptible potato cultivars.

## 1. Introduction

The potato plant (*Solanum tuberosum* L.) is susceptible to attack by many insects and pathogens. *Spongospora subterranea* f. sp. *subterranea* (Sss), the causal agent of powdery scab in potatoes, is one of the most yield-limiting pathogens of potato [1]. Powdery scab of tubers has long been considered the main disease caused by Sss infection of potato, resulting in a reduction in the quality of harvested tubers. However, it has recently been shown that the pathogen affects plant growth and tuber yields through infection of the roots [2,3,4,5]. Root infection by Sss has not been well studied compared with tuber infection. Reports from field and pot experiments indicated that root function, in terms of water and nutrient uptake, was compromised and plant growth reduced following Sss infection [2,3,6]. Inoculation of potato plants with Sss reduced the root mass in susceptible cultivars compared to tolerant cultivars in the pot trials [7]. The root infection stages of the pathogen (zoosporangia and root galling) are important, due to effects on plant productivity and because of their contribution to increased soil inoculum for the development of powdery scab (tuber disease) epidemics. Moreover, potato roots are susceptible to infection at all stages of development [8]. 

Potato root exudates play an important role in initiating interactions between the plant and soil microbes [9]. The functions of root exudates include, among others, chemotaxis induction, inhibition and stimulation of microbial growth [10], as well as influencing the colonization and activation of root-infecting pathogens [11,12] Therefore, root exudates function in the belowground plant defense [13]. The mechanisms of potato resistance to Sss root infection are not completely understood, and there is little information about the biochemical and cellular interactions of Sss with its host cells [7]. Following pathogen attack, plants, including potatoes, produce several pathogenesis and defense-related compounds, such as pathogenesis-related (PR) proteins, signal molecules, and phytoalexins, to deter the pathogen [14]. Metabolites produced in potato plants in response to pathogen attack can be identified and used as resistance biomarkers for the screening of cultivars in potato breeding programs. Molecular studies have shown that enhanced resistance is induced when plants are treated with defense-inducing agents, such as β-aminobutyric acid (BABA), thiadiazole-7-carbothioc acid S-methyl ester (BTH), and thiamine (vitamin B1), before pathogen attack [15,16]. Moreover, application of BABA has been reported to induce resistance in potatoes against Sss root infection [7] and against *Phytophthora infestans* [17,18]. Metabolomic profiling allows the detection of unknown compounds and provides functional information on the metabolic phenotypes of plants [19,20].

A comprehensive study of metabolite changes in response to Sss infection of potato roots will increase the knowledge of plant defense responses, interactions between metabolic networks, and basic plant metabolism. The primary objective of the current study was to undertake root and root exudate metabolic profiling using roots and root exudates of tolerant and susceptible potato cultivars uninoculated or inoculated with Sss. Differentially expressed metabolites were identified and quantified using ultra performance liquid chromatography coupled with quadrupole time-of-flight mass spectrometry (UPLC-Q-TOF-MS) chromatograms with unsupervised and supervised data mining methods. The study provides an unbiased quantitative view of a wide range of Sss infection-induced changes of secondary metabolites in potato roots and root exudates. A biochemical understanding of the interactions between potato and Sss is important for the development of new Sss management strategies, such as natural plant defense mechanism enhancement and the development of resistant cultivars in plant breeding programs.

## 2. Results

### 2.1. Zoosporangia Root Infection

Inoculation of plants with Sss resulted in root zoosporangia development in all 10 cultivars assessed (Table 1), while no infection was observed on the uninoculated controls of the cultivars. The root infection severity scores data confirmed the observation that none of the cultivars tested were resistant to infection of potato roots by Sss [21].

### 2.2. Determination of Metabolite Levels by Chemometric Models

**Figure 1:**
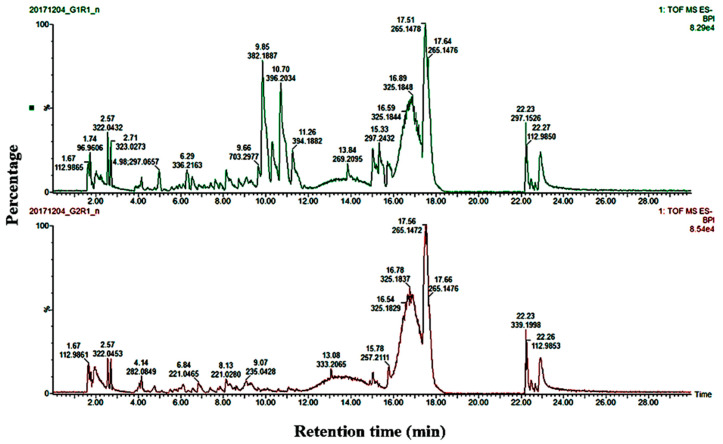
Base peak chromatogram spectra of potato root exudates collected from a tolerant potato cultivar Innovator. Represented: inoculated (G1R1; **top graph**) and uninoculated (G2R1; **bottom graph**) with *Spongospora subterranea* f. sp. s*ubterranea*.

The representative base peak intensity (BPI) of UPLC/MS chromatograms of the root exudates and roots of the two potato cultivar groups (tolerant and susceptible) are shown in Figure 1 and Figure S1, respectively. The BPI chromatograms display the complexity of the root extracts and root exudates obtained from different cultivars and show clear qualitative (presence and absence of peaks) and quantitative variation in the peak intensities for each sample. 

**Figure 2:**
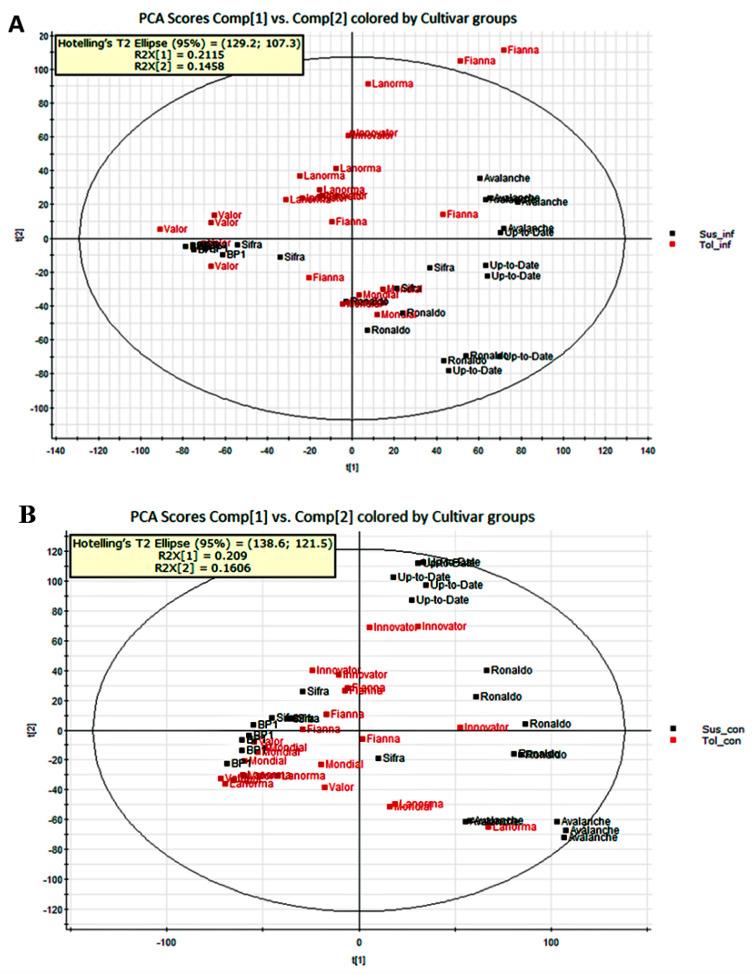
Principal component analysis plot of the ultra-performance liquid chromatography mass spectrometry data illustrating the clustering of the two cultivar groups, (**A**) five tolerant inoculated cultivars (red) vs. five susceptible inoculated cultivars (black), (**B)** five tolerant uninoculated cultivars (red) vs. five susceptible uninoculated cultivars (black). Dots represent five replications of each cultivar.

To provide comparative interpretations and visualization of the metabolic changes under different treatments, principal component analysis (PCA) was first applied to the UPLC-MS spectral datasets. A good discrimination between the tolerant inoculated and the susceptible inoculated as well as between the tolerant uninoculated and the susceptible uninoculated treatments was observed from the PCA score plots, which demonstrated that significant differences in the metabolite profiles are a result of the susceptibility or tolerance of the cultivars to Sss root infection as well as Sss inoculation and non-inoculation of the potato plants. The PCA scores plots in Figure 2 illustrate the distinct clustering of the two cultivar groups (five susceptible and five tolerant cultivars), and the similarities or differences between and within the sample clusters. 

**Figure 3:**
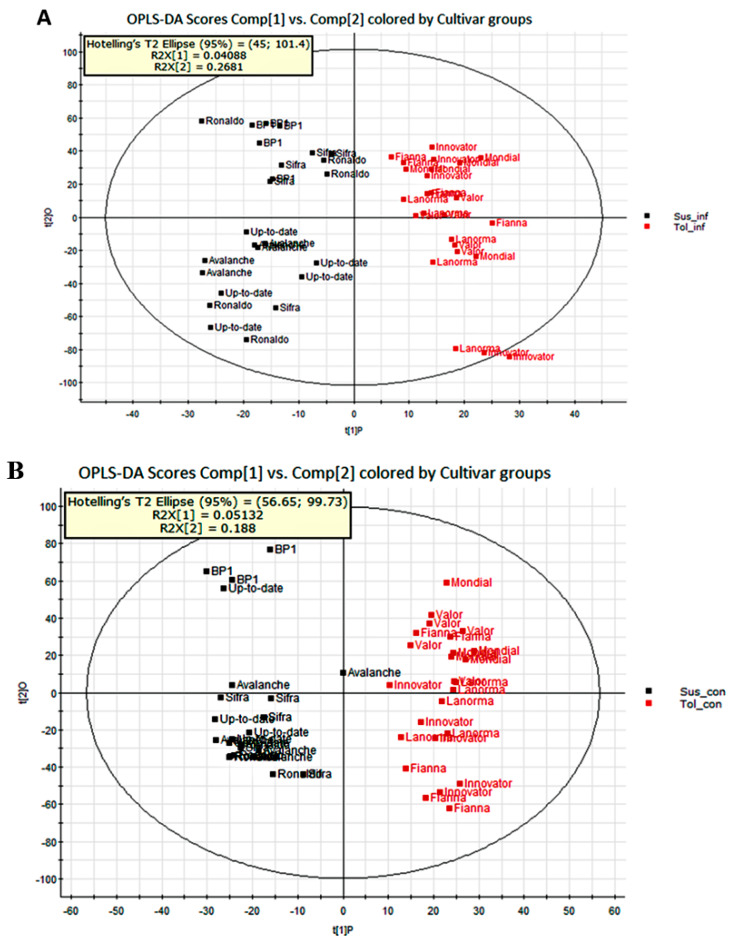
Orthogonal partial least squares discriminant analysis score plots showing a distinct separation between: (**A**) five tolerant inoculated cultivars (red) vs. five susceptible inoculated cultivars (black), (**B**) five tolerant uninoculated cultivars (red) vs. five susceptible uninoculated cultivars (black). Dots represent five replications of each cultivar.

Orthogonal partial least squares discriminant analysis (OPLS-DA) was subsequently performed to refine the separation of the two cultivar groups (tolerant and susceptible) obtained by PCA and showed clear differences in the metabolites produced between five susceptible and five tolerant cultivars. In Figure 3, the OPLS-DA scores plots show distinct sample clustering and clear cultivar separation. 

**Figure 4:**
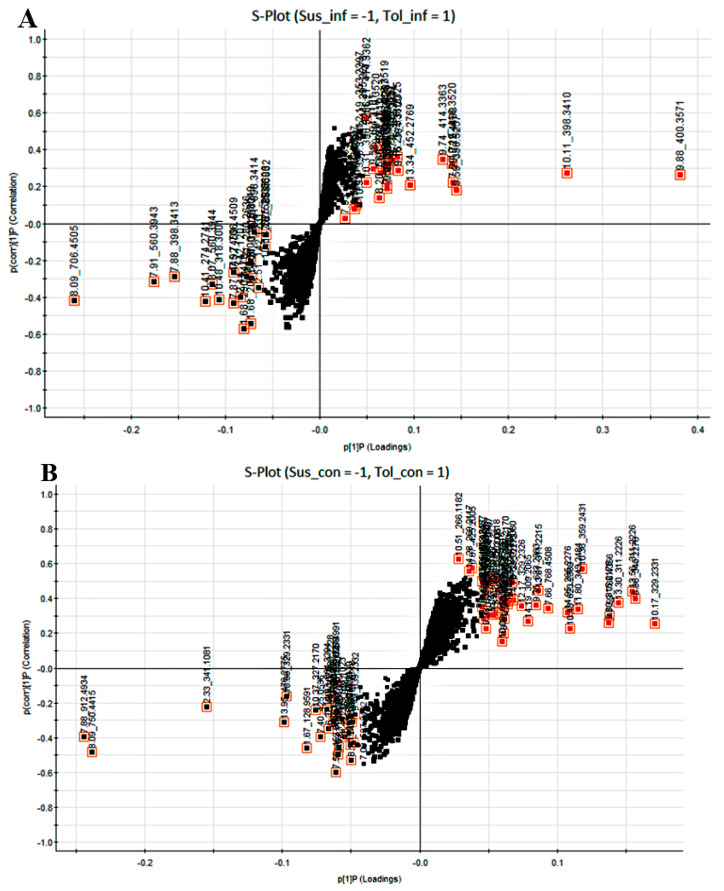
Orthogonal partial least squares discriminant analysis loadings S-plot. The markers selected in the bottom left and top right of the S-curve indicate statistically significant biomarkers identified from the orthogonal partial least squares discriminant analysis analysis and occurring predominantly in (**A**): tolerant inoculated cultivar (Tol-inf), top right vs. susceptible inoculated cultivar (Sus-inf), bottom left and (**B**): tolerant uninoculated cultivar (Tol-con), top right vs. susceptible uninoculated cultivar (Sus-con), bottom left. The black unselected squares indicate biomarkers that are common to both the susceptible and the tolerant cultivar groups.

The corresponding loading S-plots (Figure 4A,B) were used to select discriminating ions between the cultivars. The S-plots allow a visual interpretation of the OPLS-DA models to facilitate the targeting of statistically significant biomarkers. The biomarkers at the bottom left of the S-plot occur predominantly in the susceptible cultivars and those at the top right of the curve in the tolerant cultivars, respectively. The mass numbers marked with red squares (top and bottom markers) were uploaded to the online metabolite databases from MarkerLynx for putative identification of the biomarkers. 

**Figure 5:**
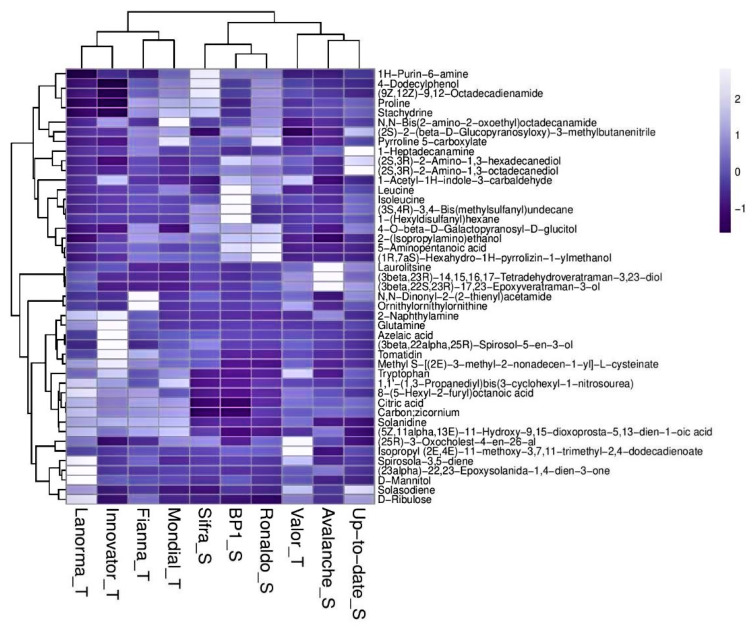
Two-way hierarchical clustering analysis visualized using a dendrogram combined with a heat map. The heat map represents the intensities of the metabolites in the roots of 10 cultivars (T = tolerant cultivar and S = susceptible cultivar).

The distribution of metabolites was further analyzed using hierarchical cluster analysis (HCA), which highlights the similarity between different cultivars based on the metabolites they contain. The computed HCA plots in Figure 5 indicate that the samples were separated into two major groups; with the tolerant cultivars clustering together and separately from the susceptible cultivars, except for one tolerant cultivar, Valor, that clustered with the susceptible cultivars. 

### 2.3. Metabolic Profiling of Potato Cultivars

Changes in the metabolites associated with Sss root infection of different potato cultivars were evaluated in this study. In order to detect changes in the expression of metabolites due to Sss infection and the susceptibility of cultivars to the pathogen, three models were built for the comparing tolerant inoculated cultivars vs. susceptible inoculated cultivars, tolerant uninoculated cultivars vs. susceptible uninoculated cultivars, and inoculated vs. uninoculated cultivar groups in positive and negative electrospray ionization (ESI) modes. 

After merging the peak intensity variables from the same metabolite, several compounds were selected as statistically significant *p* < 0.05 (Table 2; Table S1). In total, 102 and 79 major metabolites were putatively identified and selected as potential biomarkers for root exudates and potato roots, respectively. Of the 181 metabolites identified from both the roots and root exudates, only 13 metabolites were common to both the susceptible and tolerant cultivar groups (six for roots and seven for root exudates). The identified metabolites were further classified into chemical groups, such as fatty acids, sugars, sugar alcohols, amino acids, alkaloids, organic acids, and phenolics. Besides the putatively identified 181 compounds, 164 other compounds (52 from the roots and 112 from the root exudates) were detected as unknown compounds.

The most frequent and abundant metabolite groups were amino acids and alkaloids, which were both present in the highest proportion of 28%, followed by fatty acids (17%), sugars (11%), phenolics (7%), and organic acids (7%), with sugar alcohols being the least represented group with the proportion of 2%. Of the 14 amino acids identified, 5 of them, namely aminobutanoic acid, methionine sulfoxide, proline, tryptophan, and phenylalanine, were more abundant in the tolerant cultivars than in the susceptible cultivars. Six other amino acids (arginine, glutamine, kinetin, methionine, stachytrine, and 5-oxoproline) were more abundant in the susceptible cultivars, with the remaining two (leucine and valine) being equally abundant in both cultivar groups (Table 3). 

In the Sss-inoculated plants, alkaloids were upregulated in greater abundance (77%) in the tolerant cultivars than in the susceptible cultivars. Cyclopamine, laurolitsine, dehydrosolasodine, solanine, solanidane swainsonine, tomatidine, and veratramine were identified only in the tolerant cultivars, while only two alkaloids (melicopicine and trachelanthamidine) were identified only in the susceptible cultivars, and three alkaloids, namely solanidine, solasodiene, and solasodine, were common to both the susceptible and the tolerant inoculated cultivars. However, solanidine and solasodiene were significantly more abundant in the tolerant cultivars than in the susceptible cultivars (Table 3).

The results from this study highlight that of the five sugars identified, four sugars, namely D-mannitol, D-ribulose, heterodendrin, and linamarin, were significantly upregulated in the roots of the tolerant cultivars compared to the susceptible cultivars. Only nystose was significantly upregulated in the susceptible cultivars compared to the tolerant cultivars. Of the seven fatty acids identified, three were found in abundance in the susceptible cultivars compared to the tolerant cultivars, (11E) - 9, 10, 13-trihydroxy-11-octadecenoic acid, laestisaric acid, and 6-oxohexadecanoic acid. Linoleic acid amide was the only fatty acid that was common to both cultivar groups, although the tolerant cultivars had a higher concentration of linoleic acid amide than the tolerant cultivars (Table 3).

Even though the signal strength of phenolics, organic acids, and sugar alcohols was lower than the other compound groups, it is worth noting that two phenolics identified (6-hydroxymellein and quinic acid) were more abundant in the susceptible cultivars than in the tolerant cultivars. Of the three organic acids identified, the concentrations of azelaic acid and citric acid were higher in the tolerant cultivars than in the susceptible cultivars, while there was no significant difference in the abundance of quinic acid among the cultivar groups. Only one sugar alcohol, lactitol dehydrate, was putatively identified in greater abundance in the susceptible cultivars than in the tolerant cultivars (Table 3).

**Figure 6:**
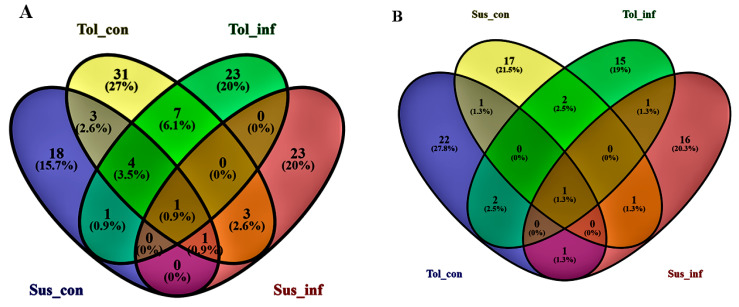
Venn diagrams displaying partial overlap of statistically significant biomarkers selected from the four OPLS-DA models comparing extracts from the (**A**) roots and (**B**) root exudates of four sample groups: Tol-con (tolerant uninoculated), Sus-con (susceptible uninoculated), Tol-inf (tolerant inoculated), and Sus-inf (susceptible inoculated). The numerical values in the diagrams depict the number of metabolites that are unique to certain cultivar groups or shared between the cultivar groups.

The Venn diagrams (Figure 6A,B) show minimal metabolite overlap in potato roots and root exudates between the four sample groups (tolerant inoculated, susceptible inoculated, tolerant uninoculated, and susceptible uninoculated). Very few of the metabolites shown in Table 2 were shared among the different cultivar groups, suggesting high metabolite variation between the tolerant and the susceptible cultivars. Figure 6A,B highlight that nine metabolites were shared between the tolerant uninoculated and susceptible uninoculated groups and only one between the tolerant inoculated and the susceptible inoculated groups in potato roots. Heterodendrin was found in all four treatment groups (tolerant uninoculated and susceptible uninoculated, as well as the tolerant inoculated and the susceptible inoculated groups) of the roots and root exudates. Four of the amino acids (arginine, solasodiene, solasodine, and valine) were present in the root exudates of both the tolerant uninoculated and the susceptible uninoculated groups, while one compound, solanidine, was present in both the tolerant inoculated and the susceptible inoculated groups.

## 3. Discussion

Plants have developed efficient mechanisms to combat pathogen attack. Timely recognition of an attacking pathogen together with the rapid and effective activation of the host defense is the main difference between resistant and susceptible plant cultivars [22,23]. The present study reports the potential application of metabolite profiling as a tool for phenotyping potato cultivars varying in susceptibility to Sss root infection.

The diversity of phytochemicals in potato roots and root exudates of five Sss tolerant and five susceptible potato cultivars was elucidated by UPLC-MS. Based on untargeted metabolomics, polar extracts (mainly amino acids, organic acids, sugars, and sugar alcohols) and nonpolar extracts (fatty acids) were putatively identified. A wide range of metabolites that play important roles in plant disease defense were abundant in the roots and root exudates of the tolerant cultivars, offering considerable scope for selecting germplasm for breeding programs. Most of the metabolites identified in the tolerant cultivars, such as D-fructose, phenylalanine, proline, and tryptophan, belonged to four metabolic pathways: The phenylpropanoid pathway, amino acid metabolism, sphingolipid metabolism, and phospholipid metabolism. These metabolites are well known for their antimicrobial properties, including cell wall thickening that is initiated at the pathogen penetration sites in response to cell wall damage [24]. Plant phenylapropanoids are involved in signal transduction, the synthesis of several defense-related metabolites, and the development of physical barriers in plants [25]. Moreover, phenylalanine is a precursor of several secondary metabolites, such as phenolics, coumarines, flavones, isoflavones, isoflavanones, lignins, tannins, and salicylic acid (SA) [26]. These metabolites are important in the defense response against pathogens and these signal molecules can activate several defense pathways, leading to more complex defense mechanisms in plants [25,27].

Alkaloids are found in potatoes in the form of glycosides of alkaloids [28]. The importance of glycoalkaloids to potato–pathogen interactions has previously been suggested [29]. The number and abundance of alkaloids was higher in the Sss-inoculated tolerant cultivars compared to the Sss-inoculated susceptible cultivars in the current study. This suggests their involvement in the defense response of the tolerant cultivars to Sss. In a similar study, the concentrations of the glycoalkaloid metabolite, solanidine, as well as of the amino acidvaline were increased in potato leaves following inoculation with *Phytophthora infestans* [26]. Moreover, increases in solanidine and solasodiene were observed in potato sprouts infected with *Rhizoctonia solani* [29]. Solanidine has been reported to inhibit pathogen infection [28] and is produced through the mevalonate pathway from acetyl coenzyme A (acetyl-CoA), which is in the cytosol, and can also be produced by the enzyme ATP-citrate lyase from the organic acid citrate [30]. Remarkably, solanidine was the most abundant metabolite identified in the roots of tolerant inoculated potato cultivars.

An organic acid, citric acid, was identified in higher amounts in the tolerant cultivars compared to the inoculated susceptible cultivars, suggesting its potential as a biomarker for Sss tolerance in potato roots. Citric acid is an intermediate of the Krebs cycle that is directly involved in the production of different amino acids belonging to the glutamic and aspartic acid families [31]. Citric acid was upregulated in the tolerant inoculated cultivars, but it was below the level of detection in the susceptible inoculated cultivars, proving its role in the defense of potato roots against Sss. Similarly, the role of citric acid in improving plant vigor against pathogen attack was also demonstrated in *Zea mays* [32]. Azelaic acid was also significantly upregulated in the tolerant cultivars in the current study. Its effect in the activation of plant defense systems was shown in tobacco through the induction of Pathogenesis-Related Proteins (PR-1a) and defensin gene transcripts [33]. PR-1a is known to act as a molecular marker for salicylic acid (SA)-dependent systemic acquired resistance (SAR), contributing to increased pathogen resistance [33], while defensin is known to have antifungal and antibacterial activities that are controlled by jasmonic acid(JA)/ethylene (ET)-dependent pathways [34].

In this study, the abundance of amino acids of the glutamic acid family, such as proline and glutamine, increased following Sss inoculation of both the tolerant and susceptible cultivars. Similarly, proline and glutamine were identified in potato root exudates of both the resistant and susceptible cultivars [9]. Increased concentrations of proline, isoleucine, leucine, and valine were found in potato leaves of both resistant and susceptible cultivars upon *Phytophthora infestans* infection, while alanine was increased only in the resistant cultivar [35]. L-proline is the main precursor in the production of cell wall proteins, such as proline-rich proteins (PRPs) and hydroxyproline-rich glycoproteins (HRGPs) [36]. Extensin, a subgroup of the HRGP family, is known for its ability to cross-link and is covalently linked to different cell wall components, such as pectin, thus increasing the mechanical strength and rigidity of the plant cell walls [37]. 

Glutamine is a shuttle for carrying nitrogen in many essential intermediate reactions in plant cells, and is a primary precursor for the production of the porphyrin ring of chlorophyll [38,39]. The results of this study suggest that glutamine is a biomarker for potato root susceptibility to Sss as its concentration was higher in the susceptible cultivars than in the tolerant cultivars. The role of glutamine in nitrogen-induced susceptibility (NIS) to the rice blast fungus in rice plants has previously been highlighted [4]. This study concluded that nitrogen fertilization increased the susceptibility of rice plants to blast due to an increase in the glutamine concentration [4]. This was due to the accumulation of the glutamine synthetase OsGS1-2 enzyme, which is responsible for the conversion of glutamate into glutamine in infected plants [4]. Moreover, glutamine was found to play an important role in stimulating Sss resting spore germination for potato root and tuber infection [9]; hence, higher glutamine concentrations were observed in the susceptible cultivars than in the tolerant cultivars. 

Interestingly, among the amino acids identified in potato roots, the non-protein 4-aminobutanoic acid (γ-aminobutyric acid, GABA) was expressed in abundance in the roots of inoculated tolerant cultivars compared to inoculated susceptible cultivars and thus, could be a potential biomarker for tolerance to Sss in potato roots. GABA is an isomer of the agrochemical β-aminobutyric acid (BABA) [7], which is used to induce resistance to Sss root infection. Some other studies on potato disease defense have demonstrated an induction of resistance with BABA treatments to *Phytophthora infestans* [16,17,18,40,41] and the necrotrophic potato pathogens *Fusarium solani* and *Fusarium sulphureum* [16,42]. The current study is the first to report GABA’s importance in potato disease defense.

Fatty acids and lipids are important sources of reserve energy, which is particularly important for the energy-intensive processes that underlie the plant defense response [43]. Lipids are also precursor molecules for the synthesis of various phytohormones, such as the fatty acid-derived JA, which has known defense gene-regulating capabilities [44].

Sugars are precursors of many metabolic pathways and are the building blocks of the cell wall’s middle lamellae, and participate in the modification of proteins and fatty acids [26]. Moreover, they are important in the production of structural defense materials, such as callose and papillae, in response to pathogen attack [26]. Similarly, most of the sugars identified in the current study were found in abundance in the tolerant cultivars compared to the susceptible cultivars, with only three identified in abundance in the susceptible cultivars.

Root exudates are comprised of low molecular-weight compounds, such as amino acids, organic acids, and phenolics [9,45], as well as high-molecular-weight compounds like proteins [46]. The current study showed that more amino acids were detected in the root exudates than in the potato roots. Similarly, higher amounts of amino acids, such as glutamine, proline, and tryptophan, were detected in potato root exudates, compared to other compound groups like the sugar alcohols, sugars, and organic acids. Root-exuded phenolics have strong antibacterial and antifungal qualities [47,48,49] and act in stimulating the chemotaxis of soil-borne beneficial microorganisms towards plant roots as well as beneficially influencing the native soil microbial community [50]. One of the organic acids identified in this study, azelaic acid, for example, has also been shown to prime plants to accumulate SA upon infection [51]. On the other hand, soil microorganisms influence the production of metabolites produced by the host in response to pathogen infection and interacting microorganisms in the rhizosphere [9,10,23]. Even though there is no scientific evidence on potato, soil type was confirmed to affect the root exudate composition of lettuce (*Lactuca sativa* L. cv. Tizian) [52]. Powdery scab has been observed in different soils in South Africa, indicating that soil type plays no role in disease development, but rather, in the severity of Sss diseases [20,53,54] and perhaps in release of metabolites.

The study demonstrated cultivar groups’ clustering, which was evident in the HCA, where all the tolerant cultivars clustered together and separate from the moderately susceptible/susceptible cultivars. However, an exception was noted with one tolerant cultivar, Valor, which grouped together with the moderately susceptible/susceptible cultivars. This suggests that some cultivars phenotypically classified as tolerant to Sss might share certain metabolic features associated with susceptible cultivars. Moreover, other compounds like the amino acids valine and proline were common to both the tolerant and the susceptible cultivars. On the other hand, among the metabolites that were uniquely upregulated in abundance in the roots or root exudates of the inoculated and uninoculated tolerant cultivars and not in the susceptible cultivars, most belonged to the phenolic and alkaloid compound groups. The current results concur with the findings from a previous study, where *Rhizoctonia solani* infection caused an increase of the phenolic compounds in resistant potato cultivars compared to the susceptible cultivar [55]. Sugars, such as D-mannintol and D-ribulose as well as 4-aminobutanoic acid, and phenylalanine belonging to the amino acid group, and the fatty acid C16 sphinganine were also uniquely found in the roots or/and root exudates of the inoculated and uninoculated tolerant cultivars and not the susceptible cultivars. This proves that these putatively identified compounds may play a significant role in plant defense against Sss infection and may be used as biomarkers for resistance in potato breeding programs.

Other than the putatively identified compounds, other compounds from both the roots and the root exudates were detected as unknown compounds; hence, further investigation using different analytical platforms, such as gas chromatography-mass spectrometry (GC-MS) and nuclear magnetic resonance (NMR) is necessary to identify the ion features that distinguish between the susceptible and tolerant cultivars in response to Sss infection.

## 4. Materials and Methods

### 4.1. Plant Growth and Spongospora subterranea f. sp. subterranea Inoculation of Plants 

Sprouted certified mini tubers of 10 potato cultivars commonly grown in South Africa (Table 4) were planted in plastic pots (13.5 cm height × 15 cm top diameter and 12 cm bottom diameter) filled with 800 g of pasteurized sandy loam soil on 15 May 2017. Ten tubers were planted for each cultivar. Plants were grown in the greenhouse at a temperature of 22 ± 2 °C with a 16 h photoperiod and were irrigated every second day with 200 mL of sterile distilled water for the maintenance of moist soil conditions favorable for root infection by Sss. Plants were fertilized fortnightly with 100 mL of solution per pot of Dr Fisher’s MultiFeed Classic (NPK 19:8:16) (Nulandis Ltd), containing trace elements. The experiment was laid out in a randomized complete block design (RCBD) with 10 cultivars, two treatments (inoculated and un-inoculated control plants), and five replicates per treatment (100 plants). 

*Spongospora subterranea* f. sp. *subterranea* inoculum was produced by removing powdery scab lesions from heavily infected field-grown tubers of BP1 cultivar using a sterile scalpel. The peels were air-dried and ground into a powder using a sterile pestle and mortar. The resulting powder was sieved through a sterile 75 μm mesh sieve. Five plants of each cultivar were inoculated at planting by mixing 4 g of Sss inoculum suspended in 50 mL of distilled water into the soil of each pot, equivalent to 5 × 10^4^ cystosori per gram of soil. The cystosorus concentration was determined using a haemocytometer. The other five plants of each cultivar were treated with 50 mL of sterile distilled water per pot to serve as the uninoculated control treatment.

### 4.2. Potato Root Exudate Collection and Root Sample Preparation

All 10 plants from each cultivar (five inoculated and five uninoculated) were gently uprooted from the soil seven weeks after emergence for root sampling and root exudate collection. For root exudate collection, roots were washed with sterile distilled water (SDW), and then blotted on sterile tissue paper. Each plant was transferred into a 750 mL polypropylene bottle (Thomas Scientific, Swedesboro, NJ 08085, USA) containing 500 mL of fresh sterile distilled water (SDW) and kept at the same temperature conditions as described for plant growth. Seven days after transferring to SDW root exudates were aseptically collected from the SDW solutions, filtered through a 2.5 µm filter paper (Whatman™ GE Healthcare UK Limited, Amersham Place UK), and stored at −20°C in the dark until metabolite extraction [9].

Immediately after collecting the root exudates, the roots were frozen in liquid nitrogen and stored at −80 °C until metabolite extraction. Roots were put in 50 mL conical Falcon centrifuge tubes and freeze-dried for two days in a Modulyo® Freeze Dryer (Thermo Electron Corporation, Waltham, MA, USA). For root sample preparation, a 100 mg sample of freeze-dried root tissue from each plant was ground into a fine powder with a mortar and pestle [49]. Root and root exudate samples were prepared from each of the 100 potato plants in the pot trial. 

### 4.3. Zoosporangia Root Infection Assessment

Another sample of roots (100 mg) was excised approximately 30 mm below the crown of each plant for evaluation of root infection by Sss zoosporangia using light microscopy. A zoosporangium staining procedure and examination under a compound microscope for the rating of zoosporangia root infection were carried out, where roots were destained in ethanol/chloral hydrate/water (1:1:1 *w/w/w*) for 10 min and then stained for 5 min in a staining solution of 3% formaldehyde, 6% lactic acid, 3.5% phenol, 87.2% ethanol/water (1:1 *v/v*), and 0.3% water blue (all *w/w*) (Merck, Darmstadt, Germany) [34]. The solution was heated to 80 °C before use. Roots were then fixed in lactic acid for five minutes, before examination under a compound microscope for root infection. A modification of the scale was used for rating the root infection from five roots per plant: 0 = no sporangia; 1 = only a few sporangia (1 to 50); 2 = several roots with little infection; 3 = several roots with moderate infection; 4 = sporangia regularly present, moderate infection; and 5 = sporangia regularly present, heavy infection [34].

### 4.4. Metabolite Extraction

In total, 100 mg of freeze-dried powdered roots and 1 mL of root exudates samples were placed in sterile 2 mL Eppendorf tubes with 1 mL of triple distilled water: methanol (30:70 *v/v*) extraction solution for roots. The mixture of dried powdered roots and 1 mL of root exudates were transferred to sterile 2 mL Eppendorf tubes, immersed in an ultrasonic bath (UMCS Ultrasonic Pty Ltd Kenware, Krugersdorp, SA), sonicated for 20 minutes at 100 W ultrasonic power, and were then centrifuged at 14,000× g for six minutes with a minispin® microcentrifuge (Eppendorf AG, Humburg, Germany). The supernatants for roots and root exudates were transferred to sterile 2 mL Eppendorf tubes and evaporated on a heat block (Accublock ™ Digital dry bath, Labnet international, Inc. Woodbridge, USA) at 60 °C overnight to remove excess solvent in the roots solution and water in the root exudates. After evaporation, the samples were cooled at room temperature for five minutes. The dry residues were re-constituted in 50:50 *v/v* acetonitrile:water solution to make up a volume of 1 mL, and the mixture was vortexed for 30 seconds. Potato root extracts were centrifuged at 14,000 × g for 5 minutes in a micro-centrifuge. The extracts were filtered through 0.22 µm filters (Whatman™ GE Healthcare UK Limited, Amersham Place UK) into pre-labelled UPLC vials fitted with slit caps (Waters, Milford, MA, USA). Metabolites were extracted for all 200 samples prepared (100 root samples and 100 root exudate samples) and all the samples were then sent to the LC-MS (SYNAPT) section of the Chemistry Department at the University of Pretoria for UPLC/MS analysis.

### 4.5. Ultra-Performance Liquid Chromatography-Time of Flight Coupled with Mass Spectrometry (UPLC–TOF-MS) Analysis

To investigate the effect of Sss infection on the phytochemical responses of potato roots, UPLC-MS (ACQUITY UPLC; Waters, Milford, MA, USA) analysis was employed to undertake metabolite profiling of 10 potato cultivars, uninoculated or inoculated with Sss. Chromatographic separation of root exudates and root extracts was performed on an ACQUITY UPLC column (HSS T3 100 mm × 2.1 mm, 1.8 µm; Waters) using mobile phase A (0.1% formic acid in deionized water) and mobile phase B (0.1% formic acid in 100% acetonitrile). Mobile phase B was isocratic at 1% until 0.2 minutes, then increased linearly to 100% at 16 minutes, and held at 100% from 16.1 until 20 minutes. Finally, solvent B was decreased to 1% at 20.1 minutes and held at 1% until 30 minutes. The injection volume of the samples was 5 µL. The column temperature was kept at 40 °C.

For mass data acquisition, the SYNAPT G2 was used in V-optics and was operated in both positive electrospray ionization-positive (ESI+) and negative (ESI-) modes. Sodium formate was used to calibrate the instrument and leucine encephalin was used as a reference calibrant to obtain typical mass accuracies. The mass spectrometer accuracy was < 0.005 Da, operated in both ESI positive and negative mode with a capillary voltage of 2.5 kV and the sampling cone voltage of 25 volts. The source and desolvation temperature were set at 120 and 400 °C, respectively. Data were acquired in MS^e^ mode, consisting of a scan using a low collision energy of 5 electron volts and a scan using a collision energy ramp from 8 to 20 volts. Helium gas was used as the nebulization gas at a flow rate of 90 mL/minute.

Stock solutions at 1 mg/mL were prepared in 50:50 *v/v* acetonitrile: water of tryptamine, sulfanilamide, mycophenolic acid, rutin, chlorogenic acid, p-coumaric acid, quercetin, cinnamic acid, and kaempferol (Sigma-Aldrich, Modderfontein, South Africa). A cocktail with each standard at 10 µg/mL was prepared and ran four times to stabilize the UPLC/MS system. A blank was injected before the samples, and the cocktail of standards was injected before the first sample, and once every eight samples to confirm system stability.

### 4.6. Multivariate Statistical Analysis

The UPLC–ESI-MS multivariate data analysis of the 200 samples was done using MassLynx version 4.1 software (Waters Corporation, Milford, MA, USA) with an added statistical program, in which the ESI positive and negative raw data were extracted and analyzed. Primary data were analyzed by Markerlynx XS™ software (Waters Corporation, Milford, USA) for alignment, peak finding, peak integration, and retention time (Rt) correction, with parameters as follows: Rt range of 1–27 min, mass range of 100–1000 Da, mass tolerance of 0.02 Da, and Rt window of 0.2 min. Data was normalized to the total intensity (area) using Markerlynx. For qualitative visualization, isotopic peaks were excluded from the analysis but included for quantitative and identification purposes. The dataset obtained from MarkerLynx™ processing was exported to the SIMCA-P software version 12.0 (Umetrics, Umea, Sweden) program in order to perform principal component analysis (PCA) and orthogonal to latent structures discriminant analysis (OPLS-DA) models, and Pareto scaling was used for both models, as well as ClustVis, a web tool for hierarchical cluster analysis (HCA). Multivariate statistical approaches, such as PCA, HCA, and OPLS-DA, were employed to explore the relationships between metabolites in order to detect differences between the tolerant and susceptible cultivars. For metabolite identification, selected markers of the loadings data from the S-plot that were accompanied by the OPLS-DA score plot were transferred to the MarkerLynx data viewer with the metabolite features of retention times and *m/z* ratios. The following identification parameters were applied using the display options: Mass search window was set at 0.1, and maximum hits were set at 50 to search for a molecular formula. Molecular markers were putatively identified at level 4 [56], based on the pseudo-molecular ions data [(M-H)^−^ or (M+H)^+^]. The putative metabolic biomarkers were identified using the MarkerLynx XS online databases and libraries, such as NIST, KEGG, ChemSpider, BioCyc, PubChem, PlantCyc, Golm metabolome, and CHeBI and LifeChemicals. The searching of these molecular formulas was restricted to C, H, N, O, S, and P elementary compositions. Data were then exported and subjected to the Student’s t-test (*p* ≤ 0.05) statistical analysis in the Statistical Analysis Software computer program (SAS 9.4; SAS Institute, 2006, Cary, NC). Data on the root infection severity were also analyzed by one-way ANOVA using SAS 9.4. Treatment means were compared using Fisher’s protected least significant difference (LSD) test at the 5% level of significance. 

## 5. Conclusions

Untargeted metabolomics analysis tentatively identified several metabolites related to the infection of potato roots by Sss. Significant differences between the tolerant and susceptible cultivar groups were identified in the levels of several metabolites, including amino acids, organic acids, alkaloids, phenolics, and sugars. These secondary metabolite classes play important roles in plant defense. The dissimilarities in the metabolic profiles and metabolites identified in the current study indicate that untargeted metabolomics can be used to distinguish between cultivars with differential levels of susceptibility to Sss. This study also illustrated that Sss infection of potato roots leads to differential expression of metabolites in tolerant and susceptible potato cultivars. The data of the present work will be useful in the documentation of a comprehensive biomarker list for use in the screening of potato breeding lines for the development of resistant potato cultivars to Sss root infection. The results indicate that the presence or absence of specific metabolites is not the only determining factor in tolerance to Sss, but the relative concentrations or ratios of metabolites between cultivars also play an important role in conferring a tolerance phenotype in potato cultivars.

## Figures and Tables

**Table 1:** Zoosporangia root infection severity of 10 potato cultivars inoculated with *Spongospora subterranea* f. sp. *subterranea.*

Cultivar	Root Infection Severity	
**s-BP 1**	3.6 *	^a^
s-Up-to-date	3.0	^ab^
t-Fianna	2.6	^ab^
s-Avalanche	2.4	^ab^
t-Innovator	2.0	^bc^
s-Ronaldo	2.0	^bc^
s-Sifra	2.0	^bc^
t-Lanorma	1.6	^bc^
t-Mondial	1.6	^bc^
t-Valor	0.8	^c^
**LSD**	1.5	
**Standard error**	0.6	

* Values are the means of five biological replicates for each cultivar. Means followed by the same letter(s) in a column are not significantly different at *p* ≤ 0.05 (LSD test).

**Table 2:** Metabolites putatively identified in roots and root exudates from potato cultivars differing in susceptibility to *Spongospora subterranea* f. sp. *subterranea* detected using UPLC-MS.

Putative Identity	ESI *	m/z	Rt (min)	Roots	Root Exudates	Tolerant Inoculated	Tolerant Un-Inoculated	Susceptible Inoculated	Susceptible Un-Inoculated
**Sugars**									
D-mannitol	+	181.0716	1.94	+			+		
D-ribulose	−	149.0454	2.34	+		+			
Heterodendrin	+	262.1291	2.57	+	+	+	+	+	+
Linamarin	+	248.1132	2.01	+			+		
Nystose	−	341.1081	2.33	+			+	+	
**Sugar alcohol**									
Lactitol dehydrate	−	343.1237	2.15	+				+	
**Amino acids**									
Arginine	−	175.1197	1.85		+		+	+	+
4-Aminobutanoic acid	−	102.0556	2.17	+		+			
Glutamine	−	145.0617	1.85	+				+	
Kinetin	+	136.0623	2.58	+				+	
Leucine	−	130.0870	2.66	+	+	+	+		+
Methionine	−	150.0603	2.76		+	+			
Methionine sulfoxide	−	166.0543	4.40		+	+			
5-Oxoproline	−	130.0506	3.95		+			+	
Phenylalanine	−	166.0846	4.77		+	+			
Proline	+	116.0711	2.15	+	+	+		+	
Pyrroline 5-carboxylate	+	130.0504	1.84	+			+		
Stachydrine	+	144.1025	2.63	+	+		+		
Tryptophan	−	203.0820	5.55	+	+	+	+		
Valine	−	118.0869	2.82		+	+	+		+
**Fatty acids**									
(6Z)-6-Decosenamide	+	338.3443	6.22		+	+	+		
Linoleic acid amide	+	280.2636	11.55	+			+		+
Laestisaric acid	−	295.2276	14.95	+					+
Octanoic acid	−	145.1259	2.73		+	+			
16-Oxohexadecanoic acid	−	269.2117	14.28	+					+
C16 sphinganine	+	274.2741	10.41	+	+	+			
(11E)-9,10,13-Trihydroxy-11-Octadecenoic acid	+	329.2329	10.98	+				+	
**Alkaloids**									
Cyclopamine	+	706.4505	8.09	+		+	+		
Dehydrosolasodine	+	398.3254	9.93	+		+			
Laurolitsine	+	314.1389	8.91	+			+		
Melicopicine	−	328.1187	7.45	+			+		+
Solanine	+	722.4457	7.59	+			+		
Solanidine	+	398.3412	10.10	+	+	+	+	+	
Solanidane	+	383.6895	9.10	+		+			
Solasodiene	+	396.3255	10.31	+	+	+	+	+	
Solasodine	+	414.3361	8.53	+	+	+	+	+	
Swainsonine	+	174.1113	2.74		+		+		
Trachelanthamidine	+	142.1207	2.44	+	+			+	+
Veratramine	+	410.3049	9.00	+		+			
Tomatidine	+	416.3519	11.28	+		+	+		
**Organic acids**									
Azelaic acid	−	187.0971	8.26	+		+		+	+
Citric acid	−	191.0194	2.58	+		+			
Erythronic acid	+	136.1032	2.72	+					+
**Phenolics**									
p-coumaric acid	+	165.0557	2.91		+		+		
6-Hydroxymellein	+	193.0501	7.63	+					+
Quinic Acid	−	191.0536	2.22	+			+		

+ The compound was present in abundance (high peak intensity) in the sample. Blank means that the compound was not abundant in the sample. * Electrospray ionization. *m/z*: mass-to-charge ratio Rt (min): Retention time in minutes.

**Table 3:** Statistically significant differences in the regulation of metabolites identified in the roots of 10 inoculated potato cultivars differing in susceptibility to *Spongospora subterranea* f. sp. *subterranea* root infection, detected using UPLC-MS.

Putative Identity	m/z	*P*-Value ^a^	Fold-Change ^b^
**Sugars**			
D-mannitol	181.0716	0.0426*	2.43
D-ribulose	149.0454	0.0272*	1.68
Heterodendrin	262.1291	0.0100**	2.17
Linamarin	248.1132	0.0049**	3.17
Nystose	341.1081	0.0229*	0.62
**Amino acids**			
4-Aminobutanoic acid	102.0556	0.0086**	3.58
Glutamine	145.0617	0.0141*	0.74
Kinetin	136.0623	0.0198*	0.63
Phenylalanine	166.0846	0.0331^*^	1.58
Proline	116.0711	0.0393*	1.67
Tryptophan	203.0820	0.0019**	2.56
Valine	118.0869	0.0024^*^	1.50
**Fatty acids**			
Linoleic acid amide	280.2636	0.0315*	1.75
Laestisaric acid	295.2276	0.0275*	1.58
C16 Sphinganine	274.2741	0.0102**	0.55
**Alkaloids**			
Solanidine	398.3412	0.0003***	3.11
Solanidane	368.6895	0.0145*	2.04
Solasodiene	396.3255	0.0092**	2.87
Trachelanthamidine	142.1207	0.0473*	0.41
Veratramine	410.3049	0.0091**	4.15
Tomatidine	416.3519	0.0025**	4.91
**Organic** acids			
Azelaic acid	187.0971	0.0136*	1.72
Citric acid	191.0194	0.0017**	2.04
**Phenolics**			
Quinic Acid	191.0536	0.0225*	0.52

^a^*p*-values calculated using the student t-test showing significant differences between the mean peak areas of the tolerant and susceptible cultivar groups. The asterisks indicate significant differences between the two cultivar groups (* *p* ≤ 0.05; ** *p* ≤ 0.01; *** *p* ≤ 0.001). ^b^ Fold-change = mean peak areas of tolerant cultivars divided by mean peak areas of susceptible cultivars. Fold change >1.5 shows the compound is upregulated in tolerant cultivars. Fold change <1.5 shows the compound is downregulated in tolerant cultivars.

**Table 4:** List of 10 potato cultivars differing in susceptibility levels to *Spongospora subterranea* f. sp. *subterranea* root infection used in pot trials.

Cultivar	Root Infection Response *
Fianna	Tolerant
Innovator	Tolerant
Lanorma	Tolerant
Mondial	Tolerant
Valor	Tolerant
Avalanche	Susceptible
Sifra	Susceptible
BP1	Susceptible
Ronaldo	Susceptible
Up-to-date	Susceptible

* Potato cultivars were arbitrarily categorized into tolerant and susceptible according to their root infection severity scores [41].

## Supplementary Materials

Supplementary materials can be found at https://www.mdpi.com/1422-0067/21/11/3788/s1.
