# Supplementary material for: Metabolomic Fingerprinting of Potato Cultivars Differing in Susceptibility to Spongospora subterranea f. sp. subterranea Root Infection
Source: Int J Mol Sci. 2020 May 27;21(11):3788. doi: 10.3390/ijms21113788 (PMC7312161; doi:10.3390/ijms21113788)
Supplement: Supplementary file 1 [file ijms-21-03788-s001.pdf]

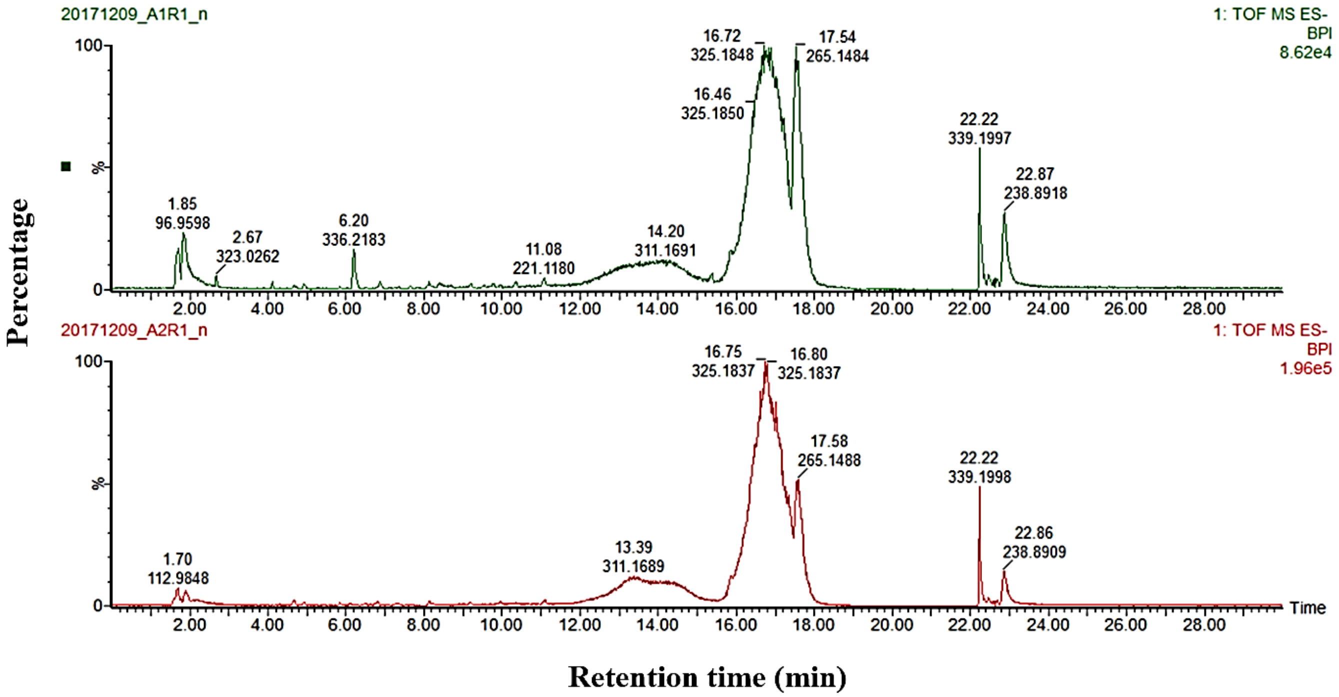

Supplementary Figure 1. Base peak chromatogram spectra of potato roots collected from a susceptible potato cultivar, Up-to-date. Represented: inoculated (A1R1; top graph) and un-inoculated (A2R1; bottom graph) with *Spongospora subterranea* f. sp. *subterranea*..

Supplementary Table 1. Organic compounds in roots and root exudates from potato cultivars differing in susceptibility to *Spongospora subterranea* f. sp. *subterranea* detected using UPLC-MS.

Putative identification	ESI*	M/z	Rt (min)	Roots	Root exudates	Susceptible cultivars	Tolerant cultivars
Dipentyl sulphite	-	221.12	11.08		+	+	
Cerulenin	-	224.13	3.83		+	+	
8-Quinolinol	+	146.06	5.54		+		+
1-Octanamine	+	130.16	5.89		+	+	
Indoline	-	120.08	4.77	+	+	+	+
Isocyanatocyclohexane		126.09	2.74		+		+
Lauraldehyde	-	185.19	7.16		+		+
2-Allyl-4(2-methyl-2butanyl)phenol	-	205.16	11.78		+		+
N,N,N,N-Tetramethylphosphonous diamide	-	121.08	4.79		+	+	+
6-Ethoxy-1,3-benzothiazole-2(3H)-thione	-	212.02	10.37		+		+
Bis(2-ethylhexyl)1,2-cyclohexanedicarboxylate	-	397.33	10.1		+		+
Phenylbis(diethylamino) phosphine	-	253.18	10.8		+		+
3,20-Bis (dimethylhydrazone)pregn-4-ene	-	399.35	10.04		+		+
Estralutin	+	429.3	13.36		+		+
6-Propyl -3,4,5-petrahydro-3-pyridinol	+	142.12	2.74		+		+
7-Cyano-7-deazaguanine	-	176.06	2.27		+		+
Cyclopamine	-	412.32	8.56		+		+
4-(Dodecylamino)-4-oxo-3-sulfobutanoic acid	-	366.19	9.88		+		+
Methysergide	-	354.22	7.27		+		+
Octylphenol	-	207.17	8.98		+		+
6-Aminohexanoic acid	-	132.1	2.74		+		+
3-Oxododecanoic acid	-	213.15	13.61		+		+
1-(2-Methoxyphenyl)piperazine	-	193.13	8.39		+		+

6-Azido-1-hexanamine	-	143.13	2.74		+	+	+
2,3,4,4-Tetrahydroxybutanoic acid	-	153.04	2.7		+		+
4-(3-Pyridinyl)butanoic acid	-	166.07	5.01		+		+
3,5-Dithiaheptane	-	137.05	2.74		+		+
4-vinylguaiacol	-	151.08	8.65		+		+
1,1'-(1,6-Hexanedyl)bis(1-nitrosourea)	+	254.15	5.14		+		+
N-Acetyleucine	+	173.11	2.52		+		+
6-Ethoxy-1,3-benzothiazole-2(3H)-thione	+	211.3	10.36		+		+
N-(3-(aminomethyl)benzyl)-acetamidine	+	177.25	5.36		+		+
Hypoglycine acid	-	142.09	1.98		+		+
4-(3-Pyridinyl)butanoic acid	-	166.09	4.77		+		+
Dethiobiotin	-	215.14	4.4		+		+
4-(Dodecylamino)-4-oxo-3-sulfobutanoic acid	-	366.2	9.88		+		+
8-Amino-7-oxononanoic acid	-	188.13	2.69		+		+
1-Acetyl-1H-indole-3-carbaldehyde	-	188.07	5.48	+	+		+
Ally bis(1-aziridinyl)phosphinate	-	188.07	5.48		+		+
2-(Phenylsulfanyl)cyclododecanone	-	291.17	6.17		+		+
(9Z)-1,9,16-Heptadecatriene-4,6-dyn-3-one	-	241.16	4.24		+		+
3,20-Bis(dimethylhydrazono)pregn-4-ene	-	399.34	10.02		+		+
Diethylene glycol, amino, N-octyl	-	218.21	7.77		+		+
Methysergide	-	354.22	7.26		+		+
Obscuraminol acid	+	278.25	13.4		+	+	
Farnesyl acetone	+	263.24	14.24		+	+	
Trimethylolmelamine	-	217.11	4.74		+	+	
Queuosine	-	410.17	11.64		+	+	
Diprogulic acid	-	275.11	5.25		+	+	
Monocyclohexyl phthalate	+	249.11	11.37		+	+	
Lauric monoethanolamide	+	244.23	8.28		+	+	
2-[2-(Octylamino) ethoxy]ethanol	+	218.21	7.78		+	+	

Heptaethylene glycol	+	327.2	5.75		+	+	
Drofenine	+	318.24	13.4		+	+	
6-Amino-2(1H)-pyrimidinone	+	112.06	2.64		+	+	
Hexadecanodoale	+	265.2	13.14		+	+	
Linoleohydroxamic acid	+	280.26	13.88		+	+	+
P,P-Di-1-piperidinylphosphinic hydrazide	+	247.17	4.24		+	+	
2-Amino-1,3,4,5-icosanetetrol	+	362.33	10.33		+	+	
N-Benzyloctadecan-1-amine	+	360.36	14.25		+	+	
2-Amino-1,3-octadecanediol	+	302.31	11.37		+	+	
Dihexylphthalate	+	335.22	13.14		+	+	
11-Aminoundecanoic acid	+	202.18	2.72		+	+	+
1,3,5-Benzenetriamine	+	124.09	4.25		+	+	
9,12,15-Octadecadrienoic acid	+	279.23	13.63		+	+	
6-Azido-1-hexanamine	+	143.13	2.73		+	+	+
6-Phenyl-4 hexyn-2-cn	+	158.244	2.72		+	+	
Betonicine	+	159.19	1.98		+	+	
2-Methoxy-4-vinylphenol	+	150.18	8.67		+	+	
N,N,N,N-Tetramethylphosphonous diamide	+	253.18	4.77		+	+	+
Dopamantine	+	315.41	4.64		+	+	
L-Leucyl-L-leucyl-aspartic acid	-	360.21	4.7		+	+	+
N-Cyclododecyl-2-(4-methoxyphenoxy) acetamide	-	348.25	7.17		+	+	+
p-Heptyloxybenzylidene p-heptylaniline	+	394.31	9.2	+			+
2-Naphthylamine	+	144.08	5.84	+			+
Glutarylcarnitine	+	276.14	2.59	+			+
6-Ethoxybenzothiazolethiol	+	212.02	10.47	+			+
4,6-Nonanedione	+	155.11	8.83	+			+
Alafosfalin	+	209.1	1.97	+			+
Dimethylphosphoramidocyanidic acid	+	133.01	2.57	+			+
Inosine	-	267.07	2.22	+			+

2,3-Dinor-8-iso PGF1α	-	327.22	9.41	+			+
Threonic acid	-	136.1	2.72		+	+	
N-Undecanoylglycine	+	242.17	10.14	+			+
8-(5-Hexyl-2-furyl) octanoic acid	+	295.23	12.11	+		+	+
15-Keto-PGE2	+	394.31	9.15	+			+
1-acetyl-3-formylindole	+	188.07	5.44	+			+
Methoprene	+	311.26	13.05	+			+
Docosapentaenoylcarnitine	+	474.36	13.5	+			+
1-18:2-lysophosphatidylethanolamine	+	478.29	13.68	+			+
Ornithylornithylornithine	-	359.24	10.42	+			+
Medemo	+	210.08	6.57	+		+	
Ureidopropanoic acid	+	131.05	1.78	+		+	
1,2-Dihydroaceanthrylene	+	203.08	5.51	+		+	+
N-[2-(Dimethylamino)propyl]hydrazinecarbothioamide	+	175.1	7.24	+		+	
2,2'-(Ccyclohexylphosphinediyl)dipyridine	-	281.21	14.37	+		+	
D-Gglucose diethyl dithioacetal	+	285.08	8.32	+		+	
Pyrroline hydroxycarboxylic acid	-	128.03	3.87	+		+	
(+)-Jasmonic acid	+	209.12	13.97	+		+	
Glycerophosphoglycerol	+	245.04	1.985	+		+	
2-(isopropylamino)ethanol	+	104.11	1.81	+		+	
5-Aminopentanoic acid	+	118.09	1.99	+		+	
1-Heptadecanamine	+	256.3	12.42	+		+	
Hexyl disulphide	+	235.16	5.88	+		+	
N,N-Dinonyl-2-(2-thienyl) acetamide	+	394.31	10.48	+		+	
Vorinostat	+	398.24	13.01	+		+	
2,3-Dinor-8-iso PGF1α	-	685.36	13.33	+		+	
Carbon;zicornium	-	128.96	1.68	+		+	
4-Dodecylphenol	+	263.24	14.21	+		+	
N-Benzyl-llinoliamide	+	280.26	14.21	+		+	

Phenylpyruvic acid	+	164.16	2.91	+	+	
4-Hydroxy-4-(3-pyridinyl)butanoic acid	+	181.19	2.91	+	+	
Ethosuximide	+	141.17	1.98	+	+	
1H-Purin-6-amine	-	136.06	2.74	+		+
Spiroxamine	+	298.28	12.61	+	+	+
Guanine	-	152.06	4.17	+	+	+
Leucylproline	+	229.16	5.16	+	+	+

*Electrospray ionization
+ The compound was present in the sample
Blank – the compound was not present in the sample
M/z-Atomic mass
Rt(min)-Retention time in minutes
